# Circular RNA circABCC4 as the ceRNA of miR‐1182 facilitates prostate cancer progression by promoting FOXP4 expression

**DOI:** 10.1111/jcmm.14477

**Published:** 2019-07-03

**Authors:** Changkun Huang, Huanghao Deng, Yinhuai Wang, Hongyi Jiang, Ran Xu, Xuan Zhu, Zhichao Huang, Xiaokun Zhao

**Affiliations:** ^1^ Department of Urology The Second Xiangya Hospital, Central South University Changsha P.R. China

**Keywords:** circABCC4, FOXP4, migration, proliferation, prostate cancer

## Abstract

In recent years, circular RNAs (circRNAs) have been identified to be essential regulators of various human cancers. However, knowledge of the functions of circRNAs in prostate cancer remains very limited. The correlation between circABCC4 and human cancer is largely unknown. This study aims to investigate the biological functions of circABCC4 in prostate cancer progression and illustrate the underlying mechanism. We found that circABCC4 was remarkably up‐regulated in prostate cancer tissues and cell lines and promoted FOXP4 expression by sponging miR‐1182 in prostate cancer cells. CircABCC4 knockdown markedly suppressed prostate cancer cell proliferation, cell‐cycle progression, migration and invasion in vitro. Furthermore, silencing of the circRNA also delayed tumor growth in vivo. Taken together, our findings indicated that circABCC4 facilitates the malignant behaviour of prostate cancer by promoting FOXP4 expression through sponging of miR‐1182. The circABCC4–miR‐1182‐FOXP4 regulatory loop may be a promising therapeutic target for prostate cancer intervention.

## 1 INTRODUCTION

Prostate cancer is the most common cancer among men in industrialized nations and a leading cause of cancer‐related death.^1^ The incidence of prostate cancer is growing rapidly throughout the world; in fact, 180 890 new cases and 26 120 deaths were reported in the United States in 2016.^2^ However, no standard methods for prostate cancer prevention, early diagnosis, treatment, or prognosis determination are yet available.^3^ Moreover, treating this disease is difficult, and the survival rate of patients is low.^4^ Currently, the most efficient hormone therapy can only achieve a median progression‐free time of 2 years.^5^ Thus, the mechanisms of prostate cancer progression must be understood.

Circular RNAs (circRNAs) were first considered splicing errors.^6^ However, developments in whole transcriptome sequencing technology have recently identified a large number of circRNAs, which are a group of endogenous non‐coding RNAs without a 5’ cap or a 3’ poly A tail.^7^ Increasing evidence indicates that circRNAs participate in many biological processes^8^, ^9^, ^10^ through diverse mechanisms, including sponging of miRNA function, thereby affecting splicing and translation.^11^, ^12^, ^13^ Dysregulated circRNAs have been reported to regulate cancer progression,^14^, ^15^ but their role in prostate cancer progression remains unknown. Thus, in this work, the function of the circRNA circABCC4 in prostate cancer is explored.

This study investigates the biological function of circABCC4 in promoting prostate cancer progression. CircABCC4 (circBase ID: hsa_circ_0030586; chr13:95813442‐95840796) was derived from the mRNA back‐splicing of ABCC4, which is located in chromosome 13q32.1. CircRNA array analysis showed that a novel circRNA named circABCC4 is highly up‐regulated in prostate cancer tissues. Moreover, high circABCC4 expression is associated with a poor prognosis in prostate cancer patients. Loss‐ or gain‐of‐function analyses indicated that circABCC4 promotes prostate cancer progression in vitro and in vivo. CircABCC4 mechanically sponges miR‐1182 expression to promote FOXP4 expression and, consequently, accelerates tumor progression. Collectively, our findings reveal that circABCC4 plays a pivotal role in prostate cancer progression.

## 2 MATERIALS AND METHODS

### 2.1 Patient specimens

A total of 47 cases of prostate cancer tumor tissues and paired adjacent normal samples was collected from the Second Xiangya Hospital, Central South University. The procedure of the present study was approved by the Institutional Review Board of the Second Xiangya Hospital, Central South University. Written informed consent was obtained from each participant prior to their participation in this research.

### 2.2 Cell culture and transfection

The prostate cancer cell lines PC3 and DU145 were obtained from the American Type Culture Collection and maintained in Dulbecco's modified Eagle's medium (Hyclone, Logan, UT, USA) supplemented with 10% fetal bovine serum, 100 units/mL penicillin and 100 μg/mL streptomycin. Cells were cultured in 5% CO_2_ at 37°C.

siRNA targeting circABCC4 (#1:5’‐AAAUCCAAUAGGCAUCAGAGA‐3’ and #2:5’‐AAUAGGCAUCAGAGACCCCAA‐3’) or scramble siRNAs was synthesized by Invitrogen. MiR‐1182 mimics, miR‐1182 inhibitors and negative controls were bought from by GenePharma (Shanghai, China). Cell transfection was conducted using Lipofectamine 2000 (Invitrogen, Carlsbad, CA) following the manufacturer's instruction.

### 2.3 Cell viability assay

A total of 4 × 10^3^ cells were seeded into 96‐well plates and then incubated for 24 hours. The cells were then transfected with the indicated RNA duplexes, treated with 10 μL of cell counting solution (WST‐8, Dojindo Laboratories, Tokyo, Japan) at different time points, and incubated for another 2 hours. The absorbance of the wells was measured spectrophotometrically at 450 nm.

### 2.4 Colony formation assay

A total of 500 transfected cells were seeded into 6‐well plates and cultured for 14 days. The cells were subsequently fixed with methanol and stained with 0.1% crystal violet. Then, the colony number was counted.

### 2.5 Flow cytometry analysis

A total of 1 × 10^6^ cells were harvested and fixed with ice‐cold 75% ethanol overnight. The cells were then incubated with 50 μg/mL propidium iodide (Invitrogen, CA, USA) and 50 μg/mL RNaseA (Sigma, St. Louis, MO, USA) for 45 minutes at room temperature. Thereafter, the samples were analysed using an Arial III FACS system (BD Biosciences, USA).

### 2.6 Luciferase assays

After plating on a 24‐well plate for 24 hours, PC3 and DU145 cells were transfected with the indicated transcripts using Lipofectamine 3000 (Invitrogen) and cultured for another 24 hours. Relative luciferase activity was measured using the Dual‐Luciferase Reporter Assay System (Promega, USA).

### 2.7 Migration and invasion assays

An 8.0 µm 24‐well Boyden chamber was used to determine the migration and invasion abilities of PC3 and DU145 cells. For the invasion assays, the chambers were pre‐coated with Matrigel, and 4 × 10^3^ cells in serum‐free medium were seeded into the upper level of the chamber. The full culture medium was added to the lower chamber as a chemoattractant. After 24 hours, the membrane was fixed with methanol and stained with 0.1% crystal violet. Five visual fields (200×) were randomly selected to count the invading cells. Migration assays were carried out without pre‐coating of the chambers.

### 2.8 qRT‐PCR

Total RNA was extracted using RNAiso Plus (TaKaRa, Japan), and cDNA was synthesized using the PrimeScript RT Reagent Kit (TaKaRa). The resulting cDNA was quantified by SYBR Green Real‐time PCR Master Mix (Thermo Fisher Scientific, USA) through an ABI 7500 Fast real‐time PCR system (Applied Biosystems, Foster City, CA, USA); here, small nuclear RNA U6 and GAPDH were used as internal controls. The primer sequences were as follows: GAPDH forward: 5’‐GGGAAACTGTGGCGTGAT‐3’, GAPDH reverse: 5’‐GGGTGTCGCTGTTGAAGT‐3’; circABCC forward: 5’‐TGAGTCAATTCTGAAAGCTCCG‐3’, circABCC reverse: 5’‐GGCCGACCACAGCTAACAAT‐3’; U6: forward, 5’‐CTCGCTTCGGCAGCACA‐3’, U6 reverse, 5’‐AACGCTTCACGAATTTGCGT‐3’; FOXP4 forward: 5’‐ATCGGCAGCTGACGCTAAATGAGA‐3’, FOXP4 reverse: 5’‐AAACACTTGTGCAGGCTGAGGTTG‐3’.

### 2.9 Western blot analysis

Cells were lysed with lysis buffer (20 mM KCl, 150 mM NaCl, 1% NP‐40, 1% Triton X‐100, 50 mM NaF, 50 mM Tris, 1 mM DTT, 1 mM EGTA, 1 × protease inhibitor and 10% glycerol) for 1 hour and centrifuged for 30 minutes at 4°C. Equal amounts of protein were separated on SDS‐PAGE gels and transferred to PVDF membranes. After blocking with 5% non‐fat milk, the membranes were incubated first with primary antibodies and then with secondary antibodies. Signals were detected using an ECL kit (Bio‐Rad).

### 2.10 Tumor xenograft assay

Female BALB/c (nu/nu) mice were purchased from Shanghai Slac Laboratory Animals Ltd. A total of 1 × 10^7^ cells were separately injected subcutaneously into the right flank of the mice, and tumor size was measured every week. The mice were sacrificed 4‐5 weeks later, and tumors were removed and weighed. The animal experiments were approved and carried out in accordance with the guidelines of the Animal Research Ethics Committee of the Second Xiangya Hospital, Central South University.

### 2.11 Statistical analyses

All statistical data are expressed as the mean ± SD of at least three independent experiments. Two‐tailed Student's *t* test and one‐way ANOVA were used to compare two and multiple groups, respectively. The Kaplan‐Meier method was used to draw survival curves and the log‐rank test was used to determine statistical significance. Pearson's correlation analysis was used to determine the correlations. A *P* value of <0.05 was considered to indicate statistical significance.

## 3 RESULTS

### 3.1 Relative expression of circABCC4 in prostate cancer tissues

**Figure 1:**
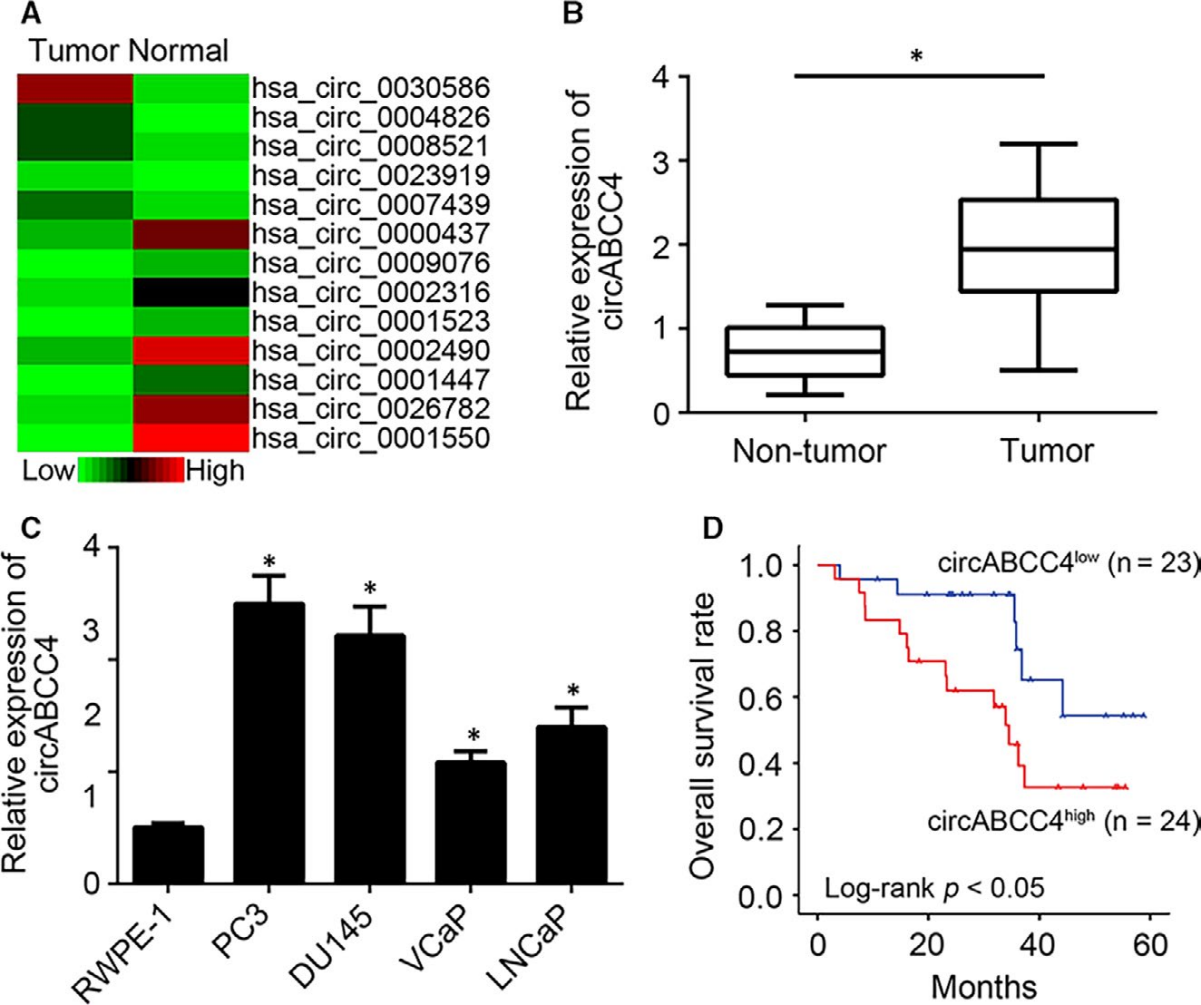
Relative expression of circABCC4 in prostate cancer tissues. A, Heatmap of differentially expressed circRNAs in pairs of prostate cancer and adjacent normal tissues according to a microarray dataset (GSE77661). B, Relative expression of circABCC4 in 47 pairs of prostate cancer and adjacent normal tissues determined by qRT‐PCR. C, Expression patterns of circABCC4 determined by qRT‐PCR in prostate cancer cell lines. D, Kaplan–Meier survival analysis based on the expression of circABCC4 in prostate cancer tissues. *P < 0.05

Although circRNAs play indispensable roles in the development of cancers,^15^ their functions in prostate cancer have not been fully investigated. Thus, circRNA expression was analysed from a public prostate cancer database (GSE77661) to reveal the possible role of circRNAs in prostate cancer carcinogenesis. Many circRNAs were differentially expressed in prostate cancer, among which circABCC4 was remarkably overexpressed (Figure 1A). CircABCC4 was also overexpressed in both prostate cancer tumors and cell lines compared with the control (Figure 1B, 1), thereby confirming that circABCC4 is overexpressed in prostate cancer. The expression of circABCC4 is correlated with prostate cancer carcinogenesis because higher circABCC4 expression indicates shorter 5‐year survival rates among patients (Figure 1D). Moreover, we found that the expression of circABCC4 was positively correlated with advanced stage and metastasis (Table 1). These findings collectively indicate that circABCC4 may be involved in prostate cancer.

**Table 1:** Correlations between circABCC4 expression and clinicopathological characteristics in prostate cancer

Characteristics	Low expression (23)	High expression (24)	*P* value
Age (years)			
≤65	10	8	0.556
>65	13	16	
Clinical T stage			
T1‐T2	15	8	0.042^a^
T3‐T4	8	16	
Lymph node metastasis			
No	17	9	0.019^a^
Yes	6	15	
Distant metastasis			
No	18	11	0.036^a^
Yes	5	13	

a *P* < 0.05 by Chi‐square test.

### 3.2 CircABCC4 deficiency inhibits prostate cancer cell proliferation, migration and invasion

**Figure 2:**
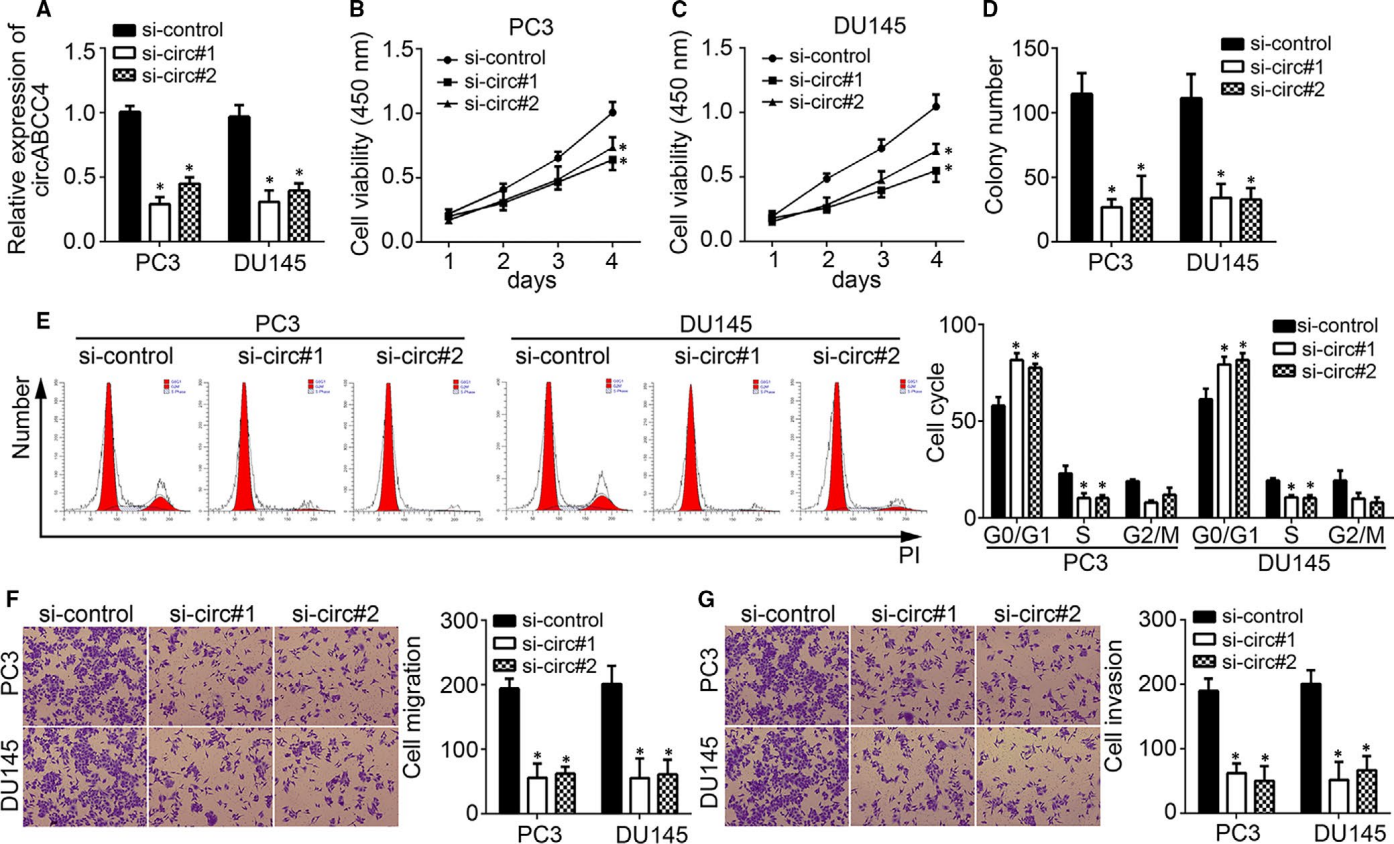
CircABCC4 knockdown inhibits prostate cancer cell proliferation, migration and invasion. A, Relative expression of circABCC4 in PC3 and DU145 cells transfected with specific siRNAs against circABCC4. B, C, CCK8 assays were used to measure cell proliferation in PC3 and DU145 cells. D, CircABCC4 knockdown reduced colony numbers. E, CircABCC4 knockdown arrested cell‐cycle progression in PC3 and DU145 cells. F, G, Transwell assay was used to determine cell migration and invasion in PC3 and DU145 cells. *P < 0.05

Functional assays were carried out to investigate the function of circABCC4 in prostate cancer progression and carcinogenesis. Because circABCC4 levels were the highest in PC3 and DU145 cell lines (Figure 1C), we chose them for further investigation. CircABCC4 expression in the PC3 and DU145 cell lines was initially depleted by siRNAs (Figure 2A), and proliferation assays were conducted. CircABCC4 deficiency inhibited PC3 and DU145 cell proliferation (Figure 2B, 2C), and only a few colonies were generated by circABCC4‐deficient PC3 and DU145 cells (Figure 2D). These phenomena are a result of blocked normal cell‐cycle progression, as circABCC4 knockdown leads to increases in the number of cells stagnating at the G0/G1 stage of the cell cycle. Thus, few cells were found in the S and G2/M stages (Figure 2E). These results show that circABCC4 promotes the proliferation of prostate cancer cells.

Carcinogenesis is characterized by the migration and invasion of cancer cells to adjacent tissue. Therefore, the role of circABCC4 in prostate cancer migration and invasion was assessed. CircABCC4 knockdown inhibited the migration and invasion of PC3 and DU145 cells (Figure 2F, 2G). Our data indicate that circABCC4 is critical for the migration and invasion of prostate cancer cells.

**Figure 3:**
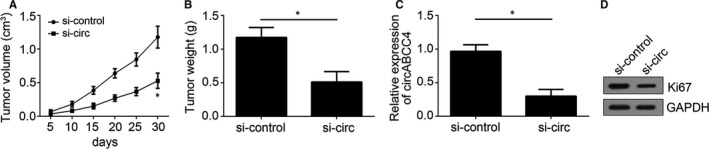
Effects of circABCC4 knockdown on cell proliferation in vivo. A, Tumor volume was measured at the indicated time points. B, Tumor weight was calculated at the end point of the xenograft experiment. C, Relative expression of circABCC4 in formed tumor tissues. D, Protein levels of Ki67 were analysed by Western blot analysis in formed tumor tissues. *P < 0.05

### 3.3 CircABCC4 promotes prostate cancer propagation in vivo

The aforementioned experiments show that circABCC4 promotes prostate cancer progression in vitro. Nude mice were injected with circABCC4‐deficient and control cells, and tumor growth was measured to confirm the function of circABCC4 in prostate cancer progression in vivo. CircABCC4 deficiency resulted in low tumor growth rates (Figure 3A, 3C) and small tumors (Figure 3B). Ki67 expression was down‐regulated in circABCC4‐deficient tumor tissues (Figure 3D) because circABCC4 regulates the proliferation of prostate cancer cell lines. These results confirm that circABCC4 promotes prostate cancer propagation in vivo.

### 3.4 CircABCC4 is a sponge of miR‐1182

**Figure 4:**
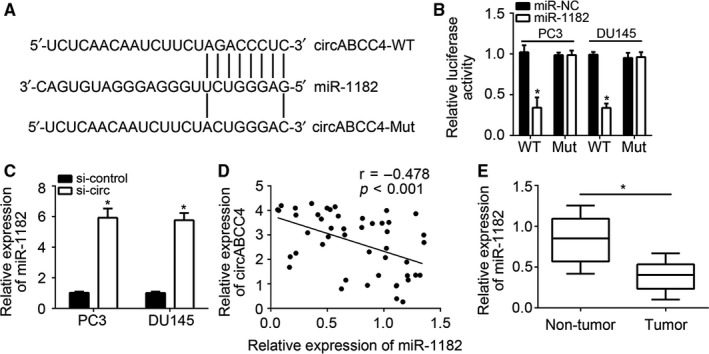
CircABCC4 is a sponge of miR‐1182. A, Predicted binding site of miR‐1182 in circABCC4. B, Luciferase reporter assay was used to verify the direct interaction between circABCC4 and miR‐1182. C, CircABCC4 knockdown promoted miR‐1182 expression in PC3 and DU145 cells. D, Correlation between circABCC4 and miR‐1182 expression in prostate cancer tissues as determined by qRT‐PCR. E, Relative expression of miR‐1182 in 47 pairs of prostate cancer and adjacent normal tissues. *P < 0.05

CircRNAs can function via miRNA binding.^16^ The possible binding partners of circABCC4 were predicted to explore the mechanism of circABCC4 in prostate cancer. An AGACCCUC motif in circABCC4 was found to match that of miR‐1182 (Figure 4A), thereby indicating the possibility that circABCC4 binds to miR‐1182. Luciferase assays showed that circABCC4 does bind to miR‐1182 (Figure 4B), and mutation of the AGACCCUC motif blocks the circABCC4–miR‐1182 interaction (Figure 4A, 4). Knockdown results showed that circABCC4 deficiency up‐regulates miR‐1182 expression (Figure 4C), which means the circABCC4–miR‐1182 interaction could inhibit miR‐1182 expression. Indeed, the expressions of circABCC4 and miR‐1182 were negatively correlated (Figure 4D), and miR‐1182 was down‐regulated in prostate cancer tissues (Figure 4E). These results are in line with the observed circABCC4 overexpression in prostate cancer and reveal that circABCC4 is a sponge of miR‐1182.

### 3.5 FOXP4 is a target of miR‐1182

**Figure 5:**
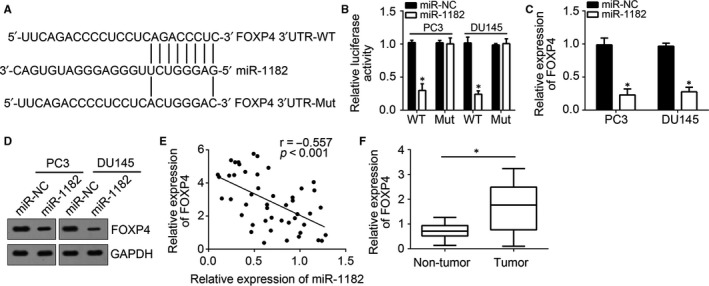
FOXP4 is a target of miR‐1182. A, Predicted binding site of miR‐1182 in the FOXP4 3’‐UTR. B, Luciferase reporter assay was used to determine the direct interaction between miR‐1182 and FOXP4 3’‐UTR. C, Overexpression of miR‐1182 suppressed the mRNA level of FOXP4 in PC3 and DU145 cells. D, Overexpression of miR‐1182 suppressed the protein level of FOXP4 in PC3 and DU145 cells. E, Correlation between miR‐1182 and FOXP4 expression in prostate cancer tissues. F, Relative expression of FOXP4 in 47 pairs of prostate cancer and adjacent normal tissues. *P < 0.05

MiRNAs can bind to and inhibit mRNA expression.^17^ To further understand the mechanism of miR‐1182 in prostate cancer progression, the binding partner of miR‐1182 was predicted, and results showed that miR‐1182 may bind to FOXP4 mRNA (Figure 5A). Luciferase experiments confirmed the binding of miR‐1182 to FOXP4 mRNA (Figure 5B). Furthermore, miR‐1182 overexpression in PC3 and DU145 cells remarkably inhibited FOXP4 expression (Figure 5C, 5D), which is in line with the negative expression correlation of miR‐1182 and FOXP4 (Figure 5E). As expected, FOXP4 expression was elevated in prostate cancer tissues (Figure 5F). Thus, miR‐1182 binds to FOXP4 and inhibits its expression, which means FOXP4 is a target of miR‐1182.

### 3.6 CircABCC4 regulates prostate cancer progression by modulating miR‐1182/FOXP4 signalling

**Figure 6:**
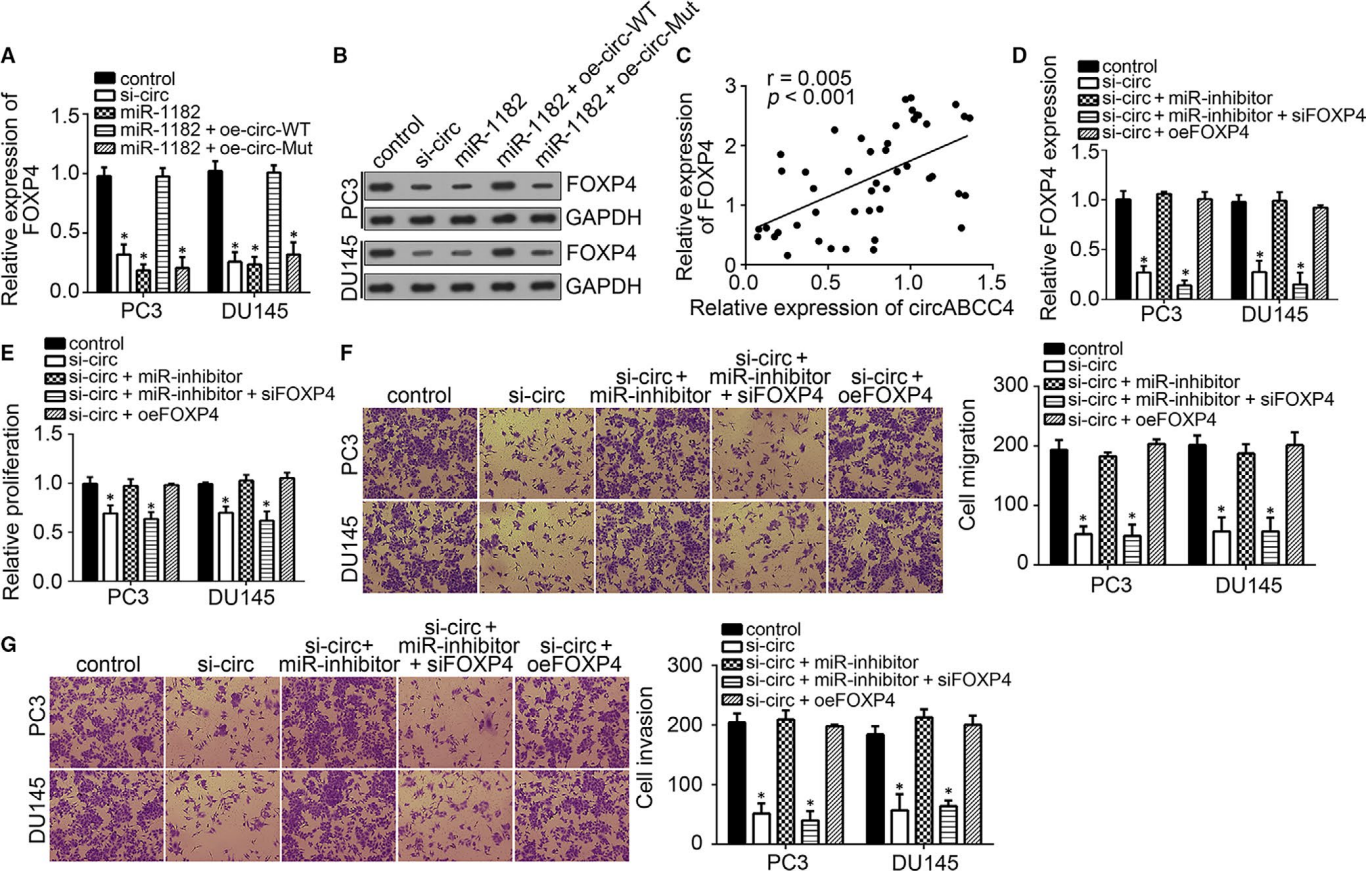
CircABCC4 regulates prostate cancer progression by modulating miR‐1182/FOXP4 signalling. A, B, Overexpression of circABCC4 abrogated the effect of the miR‐1182 mimic on FOXP4 expression in PC3 and DU145 cells, whereas mutation of the binding site in circABCC4 abolished this trend. C, Correlation between circABCC4 and FOXP4 expression in prostate cancer tissues. D, Protein levels of FOXP4 at the indicated cell lines. E, Cell proliferation was measured by CCK8 assay. F, G, Cell migration and invasion was evaluated by transwell assay of the indicated cell lines. *P < 0.05

From the results above, we can confirm that circABCC4 and FOXP4 are overexpressed whereas miR‐1182 is down‐regulated in prostate cancer. CircABCC4 also sponges miR‐1182 expression, whereas miR‐1182 negatively regulates FOXP4 expression. Studies have shown a circRNA–miRNA–mRNA regulation axis in cancer progression.^18^ Thus, we sought to determine whether circABCC4 promotes prostate cancer progression via miR‐1182/FOXP4 signalling. First, we analysed whether circABCC4 regulates FOXP4 expression and found that circABCC4 deficiency in PC3 and DU145 cells could markedly inhibit the expression of FOXP4 (Figure 6A, 6B, 6D). In addition, overexpression of circABCC4 in miR‐1182‐overexpressing PC3 and DU145 cells could rescue the inhibitory effect of miR‐1182 on FOXP4 expression (Figure 6A, 6), which means circABCC4 promotes FOXP4 expression through miR‐1182. MiR‐1182 deficiency in circABCC4‐deficient PC3 and DU145 cells remarkably rescued FOXP4 expression (Figure 6D). These results demonstrate that circABCC4 regulates FOXP4 expression through miR‐1182. CircABCC4 expression appears to be positively correlated with FOXP4 expression in prostate cancer tissues (Figure 6C), further indicating that circABCC4 regulates FOXP4 expression.

Next, we sought to discover whether circABCC4 promotes prostate cancer progression through miR‐1182/FOXP4 signalling. We inhibited miR‐1182 expression and restored FOXP4 expression in circABCC4‐deficient PC3 and DU145 cells and then conducted functional assays. Results showed that miR‐1182 inhibition in circABCC4‐deficient cells rescued the proliferation, migration and invasion defect of circABCC4‐deficient cells (Figure 6E–G). This finding indicates that circABCC4 promotes prostate cancer progression through miR‐1182. In addition, we found that circABCC4 promotes prostate cancer progression through FOXP4 because FOXP4 overexpression in circABCC4‐deficient cells could rescue the proliferation, migration and invasion defect of circABCC4‐deficient cells (Figure 6E–G).

The results thus far show that circABCC4 promotes prostate cancer progression through miR‐1182 and FOXP4 and that FOXP4 is a target of miR‐1182. We verified whether circABCC4 promotes prostate cancer progression via the circRNA–miRNA–mRNA axis by knocking down FOXP4 expression in circABCC4‐ and miR‐1182‐deficient PC3 and DU145 cells (Figure 6D) and conducting functional assays. FOXP4 knockdown greatly inhibited the proliferation, migration and invasion of PC3 and DU145 cells. Thus, circABCC4 promotes prostate cancer progression through miR‐1182/FOXP4 signalling.

## 4 DISCUSSION

CircRNAs are known to be involved in the progression of many cancers.^14^, ^15^, ^19^ Previous studies have shown that circRNAs are dysregulated in many cancers.^20^, ^21^, ^22^ Several scholars have shown that circSMARCA5 is up‐regulated in prostate cancer, and this up‐regulation promotes the progression of the disease. Despite the progress researchers have made in the field of cancer function, the function of circRNAs in prostate cancer progression remains incompletely understood, and the related mechanisms are still unclear.

In this study, circABCC4 was remarkably up‐regulated in prostate cancer tissues, and high circABCC4 expression leads to poor survival from prostate cancer. CircABCC4 deficiency dramatically inhibited PC3 and DU145 cell proliferation, migration and invasion. Thus, circABCC4 serves as an oncogene in prostate cancer progression. According to previous studies, circRNAs can sponge miRNAs to play an essential role in transcriptional control.^23^ In the present study, circABCC4 appeared to sponge miR‐1182 and promoted prostate cancer progression through miR‐1182 because miR‐1182 deficiency in circABCC4‐deficient cells greatly rescued circABCC4 deficiency‐induced prostate cancer progression defects. However, circABCC4 may also interact with other miRNAs according to bioinformatics analysis. Thus, whether other miRNAs have a role in circABCC4‐regulated tumorigenesis needs to be investigated in the future.

The circRNA–miRNA–mRNA axis has been reported to be associated with cancer progression.^23^ In this study, miR‐1182 bound to FOXP4 and inhibited FOXP4 expression. Previous studies have also demonstrated that FOXP4 is overexpressed in non‐small cell lung cancer and modulates tumor cell growth,^24^ thereby indicating that FOXP4 may function as an oncogene. One study showed that FOXP4 is involved in prostate cancer progression.^25^ In the present study, FOXP4 was overexpressed in prostate cancer, and miR‐1182 bound to FOXP4 and inhibited in expression in PC3 and DU145 prostate cancer cells. CircABCC4 regulates FOXP4 expression by sponging miR‐1182 because miR‐1182 inhibition releases the inhibitory effect of circABCC4 deficiency on FOXP4 expression. The circRNA also promotes prostate cancer progression through FOXP4 because FOXP4 overexpression could rescue the progression defect mediated by circABCC4 deficiency. Thus, circABCC4 promotes prostate cancer progression through the circABCC4–miR‐1182‐FOXP4 axis.

## 5 CONCLUSION

Taking the results together, we showed that circABCC4 expression is elevated in prostate cancer and revealed that the circRNA functions as an oncogene. CircABCC4 mechanically sponges miR‐1182 expression, resulting in the up‐regulation of FOXP4 and prostate cancer progression. We further discovered a critical circABCC4–miR‐1182‐FOXP4 axis in prostate cancer progression and demonstrated the important role of circRNAs in the progression of this disease. However, our study also has a limitation. Because we performed bioinformatics analysis to screen out circABCC4 only using a pair of tumor tissues and normal control, other important circRNAs may be ignored.

## CONFLICTS OF INTEREST

All authors declare that they have no conflicts of interest.

## AUTHORS CONTRIBUTION

CH and XZ initiated this study and designed the experiments. CH performed experiments and analysed the data. HD, YW, HJ, RX, XZ and ZH analysed the data. XZ wrote the manuscript. All authors read the manuscript and approved it.

## Data Availability

All data generated or analysed during this study are included in this published article.
